# MR arthrography is slightly more accurate than conventional MRI in detecting TFCC lesions of the wrist

**DOI:** 10.1007/s00590-018-2215-x

**Published:** 2018-04-26

**Authors:** B. C. Boer, M. Vestering, S. M. van Raak, E. O. van Kooten, R. Huis in ’t Veld, A. J. H. Vochteloo

**Affiliations:** 1 Hand and Wrist Unit, Centre for Orthopaedic Surgery OCON, PO Box 546, 7550 AM Hengelo, The Netherlands; 2 Department of Radiology, Ziekenhuisgroep Twente, PO Box 546, 7550 AM Hengelo, The Netherlands; 3 Department of Radiology, Medisch Spectrum Twente, Postbus 50 000, 7500 KA Enschede, The Netherlands; 4 Department of Plastic Surgery, Medisch Spectrum Twente, Postbus 50 000, 7500 KA Enschede, The Netherlands

**Keywords:** Triangular fibrocartilage complex (TFCC), Magnetic resonance imaging (MRI), MR arthrography, Arthroscopy, Sensitivity, Specificity

## Abstract

**Introduction:**

In case of clinical suspicion of triangular fibrocartilage complex (TFCC) injury, different imaging techniques are used. The aim of this study was to determine whether MRA is superior to MRI and whether 3.0 T is better than 1.5 T (expresses in sensitivity, specificity and accuracy) in detecting TFCC injury, using arthroscopy as the gold standard.

**Materials and methods:**

The arthroscopic and MR findings of 150 patients who underwent arthroscopy for ulnar-sided wrist pain between January 2009 and November 2016 were retrospectively reviewed.

**Results:**

MRA was slightly more accurate compared to conventional MRI, and 1.5 T was slightly more accurate than 3.0 T. 1.5 T wrist MRA had a sensitivity of 80%, a specificity of 100% and accuracy of 90%; 3.0 T wrist MRA 73, 100 and 86%, resp. Conventional 1.5 T wrist MRI had a sensitivity of 71%, a specificity of 75% and accuracy of 73%. For 3.0 T conventional MRI, this was 73, 67 and 70%, resp.

**Conclusions:**

MRA seems slightly superior to conventional MRI, but one could question whether this difference in diagnostic accuracy outweighs the burden and risks of an invasive procedure for patients with its additional costs. Furthermore, we could not confirm the superiority of 3 T compared to 1.5 T.

## Introduction

Triangular fibrocartilage complex (TFCC) injuries are a common cause of ulnar-sided wrist pain. They may lead to instability of the distal radioulnar joint with secondary deterioration of the wrist joint and functional disability [1, 2]. The TFCC is composed of the dorsal and volar radioulnar ligament, the central articular disc, meniscus homologue, ulnar collateral ligament, extensor carpi ulnaris subsheath and origin of ulnolunate and ulnotriquetral ligaments [3]. TFCC injury has been attributed to either degenerative changes or trauma.

Since physical examination and medical history do not always lead to a clear diagnosis, diagnosis of TFCC injury can be a complex and difficult clinical issue as it has a large differential diagnosis [4, 5]. However, a correct pre-operative diagnosis is important because the success of the subsequent medical intervention given will depend on it [6]. The gold standard diagnostic investigation is arthroscopy. However, due to being an invasive procedure and its costs, it is seldom used as a diagnostic tool if there is no high probability of proceeding to direct therapeutic intervention [7, 8].

Radiography can therefore be utilized in narrowing down the diagnosis for ulnar-sided wrist pain [4, 9]. Both MRI and MR arthrography (MRA) have been considered important diagnostic investigations for patients with ulnar-sided wrist pain [10]. However, due to the lack of evidence regarding the superiority of sensitivity and specificity, both MRI and MRA are used for diagnosis of TFCC injuries [11–15]. In addition, where a field strength of 1.5 T has been the reference standard for MRI of the wrist for more than a decade [16], a field strength of 3.0 T is being used since quite recently. MR field strength appears to be important, since MR images obtained at greater field strength give an increase in signal-to-noise ratio and contrast-to-noise ratio. At similar spatial resolution, this leads to an improvement in image quality and hypothesized greater diagnostic test accuracy [16, 17]. However, clinical evidence regarding the superiority of sensitivity and specificity for 1.5 and 3.0 T is lacking [11, 13, 14].

The aim of this study was to determine whether MRA is superior to MRI and whether 3.0 T is better than 1.5 T (expressed in sensitivity, specificity and accuracy) in detecting TFCC injury, using arthroscopy as the gold standard.

## Methods

Between January 2009 and November 2016, 304 patients who presented with ulnar-sided wrist pain underwent wrist arthroscopy in two large Dutch hospitals. The reason for arthroscopy in all patients was a clinical and/or radiological diagnosis of TFCC injury and persistent symptoms, despite conservative treatment (physiotherapy, brace or local injection with corticosteroids). The clinical diagnosis was based upon medical history and physical examination (tender region of TFCC, positive waiter test and/or TFCC compression test).

Of the 304 patients who underwent arthroscopy, 203 underwent a pre-operative MRI or MRA of 1.5 or 3.0 T. The strength of the MRI field (1.5 or 3.0 T) was based upon time; during the study period, the 3.0 T MRI was installed in both hospitals. MRI of MRA was chosen upon preference of the treating hand surgeon and local protocol.

Exclusion criteria for our study were: MRI or MRA performed with < 1.5 T; new trauma between MRI and arthroscopy; TFCC surgery in the past; interval between MRI or MRA and arthroscopy of more than 6 months; and a systemic joint disease (i.e. gout or rheumatoid arthritis).

Based on these criteria, 53 of the 203 patients who underwent a pre-operative MRI were excluded. Our final study population therefore consisted of 150 patients, of whom 15 patients underwent 1.5 T MRI; 105 patients underwent 3.0 T MRI; 12 patients underwent 1.5 T MRA; and 18 patients underwent 3.0 T MRA.

The 1.5 T scans were made using Siemens Avanto or Siemens Aera, both with a flex coil. The 3.0 T scans were made using Siemens Skyra scanner or Philips Ingenia, both with a dedicated wrist coil. MRA was performed after injection with diluted gadolinium in the radiocarpal joint under fluoroscopic guidance, and 5–6 ml of a mixture of 0.3 ml gadolinium (0.5 mmol/ml) in 250 ml of normal saline was used. Of all 1.5 and 3.0 T for both MRI and MRA scans, the examination protocol included coronal, sagittal and axial planes.

The initial reports of all pre-operative MRI and MRA scans and the arthroscopic findings (as described in the operative report) were reviewed.

### Statistical analysis

Demographic data of the patients were reported as mean with standard deviation or numbers with corresponding percentages as appropriate. The results of pre-operative conventional 1.5 and 3.0 T MRI and for 1.5 and 3.0 T MRA were categorized as true positive, false positive, true negative and false negative, using wrist arthroscopy as gold standard. Sensitivity, specificity, positive predicting value (PPV), negative predicting value (NPV) and area under the curve (AUC) for accuracy with corresponding 95% confidence interval (CI) were calculated accordingly. The area under the curve is a summary measure of the accuracy of a diagnostic test, i.e. how well the test separates the group being tested into those with and without the disease in question. Accuracy is classified by the traditional academic point system (0.90–1 excellent; 0.80–0.90 good; 0.70–0.80 fair; 0.60–0.70 poor; 0.50–0.60 fail) where the maximum AUC of 1 means that the diagnostic test is perfect in the differentiation between the diseased and non-diseased patients. The minimum AUC (0.5) should be considered a chance level [18].

## Results

In total, 150 patients who underwent arthroscopy for ulnar-sided wrist pain were retrospectively evaluated. Mean age of the study population was 38 ± 15 years, and 88 (59%) patients were female. History of trauma was recorded in 78 (52%) patients, and the average time between MRI or MRA and surgery was 81 ± 39 days.

**Fig. 1:**
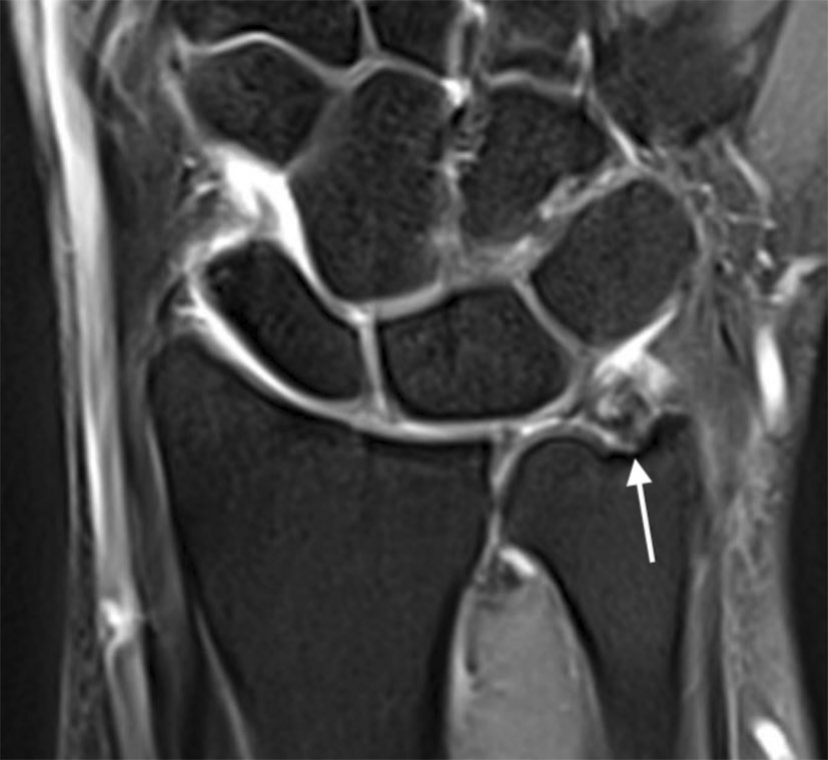
Coronal T2-weighted wrist MRI showing a TFCC tear (arrow)

**Fig. 2:**
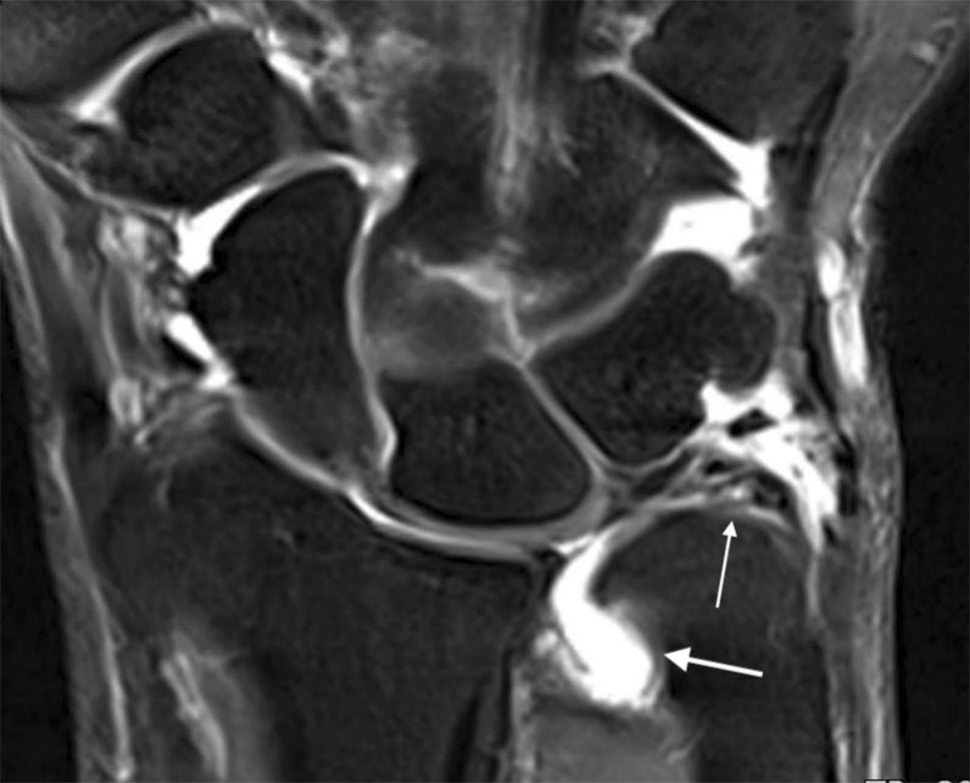
Coronal T2-weighted wrist MRA showing a TFCC tear (thin arrow) with contrast leakage (thick arrow)

A TFCC tear was found during arthroscopy in 99 of the 150 patients (66%). In 73 (73%) of those patients, the TFCC tear was correctly detected on the pre-operative MRI (Fig. 1) or MRA (Fig. 2) scan.

Our results suggest that for diagnosing TFCC injury MRA was slightly more accurate than MRI and at 1.5 T was slightly more accurate than at 3.0 T (Table 1).

**Table 1:** Sensitivity, specificity, PPV, NPV and area under the curve (AUC) with 95% CI and *p* value of 1.5 and 3.0 T MRI and MRA for TFCC injury

Imaging modality	Tear on MR	Tear on arthroscopy	Sensitivity (%)	Specificity (%)	PPV (%)	NPV (%)	AUC	95% CI interval	*p* value
MRI									
1.5 T (*n* = 15)	7	7	71	75	71	75	0.73	0.46–1.00	0.13
3.0 T (*n* = 105)	63	71	73	67	83	52	0.70	0.60–0.81	0.001
MRA									
1.5 T (*n* = 12)	8	10	80	100	100	50	0.90	0.72–1.00	0.086
3.0 T (*n* = 18)	8	11	73	100	100	60	0.86	0.69–1.00	0.011

*PPV* positive predicting value, *NPV* negative predicting value, *AUC* area under the curve, *CI* confidence interval

### MRI

A tear of the TFCC was identified on 1.5 T wrist MRI images in seven out of 15 (47%) patients, of which two patients did not have a tear on arthroscopy. Accuracy was 0.73 (95% CI 0.46–1.00), suggesting fair ability of 1.5 T MRI to correctly identify those with and without a TFCC injury.

On 3.0 T MRI images, a tear of the TFCC was identified in 63 out of 105 (60%) patients, of which 11 were false-positive. Accuracy of 3.0 T MRI was 0.70 (95% CI 0.60–0.81), suggesting fair identification of patients with and without a TFCC injury.

### MRA

On MRA, a tear was identified on 1.5 T images in eight out of 12 (67%) patients. There were no false-positive readings of TFCC tears compared with gold standard arthroscopy. The accuracy of 0.90 (95% CI 0.72–1.00) implies excellent discrimination of 1.5 T MRA between patients with and without a TFCC injury.

On 3.0 T images, a tear was identified in eight out of 18 (44%) patients. There were no false-positive readings of TFCC tears. Accuracy was 0.86 (95% CI 0.69–1.00), indicating good identification of the patients into those with and without a TFCC lesion.

## Discussion

Our study aimed to compare the diagnostic accuracy, sensitivity and specificity for both 1.5 and 3.0 T MRI and MRA compared with arthroscopy for the diagnosis of TFCC injury. The present study revealed some interesting findings. MRA was slightly more accurate compared to MRI as was 1.5 T MRI compared to 3.0 T.

In the literature, a similar trend can be found in favour of the MRA compared to MRI. However, this increase in accuracy is considerably larger than in our study [14, 19]. In comparison with the study of Moser et al. [19], the sensitivity of MRI in our study was considerably higher, whereas sensitivity of MRA was comparable. This could be an explanation for the smaller difference we found in diagnostic accuracy between MRI and MRA. Lee et al. [14] also found that 3.0 T MRA has a higher sensitivity than 3.0 T MRI. However, this is the only study where the conventional MR image was compared to an isovolumetric 3D THRIVE (T1 High-Resolution Isotropic Volume Examination) sequence MR arthrography. A meta-analysis by Smith et al. [20] shows comparable results in favour of MRA. However, not all included studies used arthroscopy as reference standard. Gupta et al. [21] suggested that arthroscopy might be superior to arthrotomy due to magnification of the wrist and allowing access to all areas of the TFCC. In addition, there is variability in the MRA technique employed (single-compartment injection technique versus a variety of combined compartment injections), as we only used single-compartment technique. Smith et al. [20] did not perform subgroup analysis, due to insufficient data. As MRA relies on the indirect assessment of a ligament by demonstrating contrast leakage, the use of these different techniques may affect correct evaluation and thus sensitivity, specificity and accuracy. Surprisingly, there is no study that investigated if there is a difference in diagnostic accuracy. While MRA seems slightly more accurate in our series, the use of this technique instead of MRI for patients with suspicion on TFCC injury entails some disadvantages: the need for injection of contrast material into the joint leads to a technically more complicated and more time-consuming procedure than conventional MRI with additional costs and the risk of infection [15, 22]. In addition, as described by Magee et al. [13], it can result in false-positive outcomes because of so-called micro-perforations of the TFCC disc.

Surprisingly, our results show that diagnosing TFCC injury at 1.5 T is slightly more accurate than at 3.0 T. However, Anderson et al. [11] concluded that sensitivity, specificity and accuracy are consistently higher for 3.0 T MRI compared to 1.5 T MRI. However, this improvement was a trend without statistical significance for only the combined evaluation of two musculoskeletal radiologists, whereas the improvement in sensitivity and specificity for 3.0 T was not present for one of the radiologists. No other studies comparing 1.5 and 3.0 T have been conducted yet. Individual studies investigating 1.5 T for diagnosing TFCC injury show sensitivity ranging from 44 to 100% and specificity from 69 to 100% [10, 15, 23, 24]. Studies investigating 3.0 T show sensitivity ranging from 60 to 100% and specificity of 74 to 100% [11, 13, 14]. An explanation for not performing better than expected of 3.0 T may be the adjustment of the settings of the scanner parameters. These are found to be more complex for the musculoskeletal system [16, 25]. During the beginning of the study period, there was no optimal protocol developed for the 3.0 T wrist MR, which might have led to a smaller improvement in image quality than expected. Also, in comparison with MRI, the majority of our MRAs were conducted in the first few years after implementation of 3.0 T in our hospital. Since there is a learning curve for interpretation of 3.0 T, this, along with the adjustment of the settings, could be an explanation for our results. We did not investigate the effect of experience of the radiologist, as subgroup analysis was not possible due to the many radiologists (and therefore low numbers of MRI per radiologist). However, this has been suggested to be a factor for variation in diagnostic accuracy of both MRI and MRA [7, 9, 26].

We have aimed to determine whether MRA is superior to MRI and whether 3.0 T is better than 1.5 T. However, as it is known that not all (untreated) TFCC injuries are symptomatic, one should keep in mind that the findings of the MR should be related to the clinical presentation [27]. In fact, when TFCC injury is clinically suspected, one could also directly perform arthroscopy without additional diagnostic investigations, to confirm the diagnosis and proceed to therapeutic intervention, as it is a safe method of treatment [28].

This study is the first report comparing diagnostic accuracy of 1.5 T MRI, 3.0 T MRI, 1.5 T MRA and 3.0 T MRA for patients who present with signs and symptoms of a TFCC injury. In comparison with previous comparable studies, our total study population is relatively large. The study is limited because of its retrospective character. Additionally, the rate of false negative outcomes is difficult to evaluate as arthroscopy was not performed in patients without symptoms.

## Conclusion

Results of the current study show that MRA seems slightly superior in terms of diagnostic accuracy. However, one could question whether this difference in diagnostic accuracy outweighs the burden and risks of an invasive procedure (as is MRA) for patients and its additional costs. Furthermore, we could not confirm the superiority in terms of sensitivity, specificity and accuracy of 3 T compared to 1.5 T in contrast to limited current evidence. More (prospective) studies are needed to confirm or refute our results of the estimated sensitivity, specificity and accuracy parameters of 1.5 and 3.0 T MRI and MRA.

## References

[1] Shih JT, Hou YT, Lee Tan CM (2000). Chronic triangular fibrocartilage complex tears with distal radioulnar joint instability: a new method of triangular fibrocartilage complex reconstruction. J Orthop Surg Hong Kong.

[2] Watson KH, Weinzweig J, Zeppieri J (1997). The natural progression of scaphoid instability. Hand Clin.

[3] Palmer AK, Werner FW (1981). The triangular fibrocartilage complex of the wrist—anatomy and function. J Hand Surg Am.

[4] Vanden Eynde S, De Smet L, Fabry G (1994). Diagnostic value of arthrography and arthroscopy of the radiocarpal joint. Arthroscopy.

[5] Schweitzer ME, Brahme SK, Hodler J (1992). Chronic wrist pain: spin-echo and short tau inversion recovery MR imaging and conventional and MR arthrography. Radiology.

[6] Garcia-Elis M, Geissler W, Green D, Hotchkiss R, Pederson W, Wolfe S (2005). Carpal instability. Green’s operative hand surgery.

[7] Blazar PE, Chan PS, Kneeland JB, Leatherwood D, Bozentka DJ, Kowalchick R (2001). The effect of observer experience on magnetic resonance imaging interpretation and localization of triangular fibrocartilage complex lesions. J Hand Surg.

[8] Oneson SR, Timins ME, Scales LM, Erickson SJ, Chamoy L (1997). MR imaging diagnosis of triangular fibrocartilage pathology with arthroscopic correlation. AJR Am J Roentgenol.

[9] Joshy S, Ghosh S, Lee K, Deshmukh SC (2008). Accuracy of direct magnetic resonance arthrography in the diagnosis of triangular fibrocartilage complex tears of the wrist. Int Orthop.

[10] Potter HG, Asnis-Ernberg L, Weiland AJ, Hotchkiss RN, Peterson MG, McCormack RR (1997). The utility of high-resolution magnetic resonance imaging in the evaluation of the triangular fibrocartilage complex of the wrist. J Bone Joint Surg Am.

[11] Anderson ML, Skinner JA, Felmlee JP, Berger RA, Amrami KK (2008). Diagnostic comparison of 1.5 Tesla and 3.0 Tesla preoperative MRI of the wrist in patients with ulnar-sided wrist pain. J Hand Surg Am.

[12] Hobby JL, Tom BD, Bearcroft PW, Dixon AK (2001). Magnetic resonance imaging of the wrist: diagnostic performance statistics. Clin Radiol.

[13] Magee T (2009). Comparison of 3-T MRI and arthroscopy of intrinsic wrist ligament and TFCC tears. AJR Am J Roentgenol.

[14] Lee YH, Choi YR, Kim S, Song HT, Suh JS (2012). Intrinsic ligament and triangular fibrocartilage complex (TFCC) tears of the wrist: comparison of isovolumetric 3D-THRIVE sequence MR arthrography and conventional MR image at 3 T. Magn Reson Imaging.

[15] Scheck RJ, Romagnolo A, Hierner R, Pfluger T, Wilhelm K, Hahn K (1999). The carpal ligaments in MR arthrography of the wrist: correlation with standard MRI and wrist arthroscopy. J Magn Reson Imaging.

[16] Saupe N, Prussmann KP, Luechinger R, Bosiger P, Marincek B, Weishaupt D (2005). MR imaging of the wrist: comparison between 1.5- and 3-T MR imaging—preliminary experience. Radiology.

[17] Haims AH, Schweitzer ME, Morrison WB (2003). Internal derangement of the wrist: indirect MR arthrography versus unenhanced MR imaging. Radiology.

[18] Hajian-Tilaki K (2013). Receiver operating characteristic (ROC) curve analysis for medical diagnostic test evaluation. Casp J Intern Med.

[19] Moser T, Dosch JC, Moussaoui A, Dietemann JL (2007). Wrist ligament tears: evaluation of MRI and combined MDCT and MR arthrography. AJR Am J Roentgenol.

[20] Smith TO, Drew B, Toms AP, Jerosch-Herold C, Chojnowski AJ (2012). Diagnostic accuracy of magnetic resonance imaging and magnetic resonance arthrography for triangular fibrocartilaginous complex injury: a systematic review and meta-analysis. J Bone Joint Surg Am.

[21] Gupta R, Bozentka DJ, Osterman AL (2001). Wrist arthroscopy: principles and clinical applications. J Am Acad Orthop Surg.

[22] Pederzini L, Luchetti R, Soragni O (1992). Evaluation of the triangular fibrocartilage complex tears by arthroscopy, arthrography, and magnetic resonance imaging. Arthroscopy.

[23] Morley J, Bidwell J, Bransky-Zaachary M (2001). A comparison of the findings of wrist arthroscopy and magnetic resonance imaging in the investigations of wrist pain. J Hand Surg.

[24] Shionoya K, Nakamura R, Imaeda T, Makino N (1998). Arthrography is superior to magnetic resonance for diagnosing of the triangular fibrocartilage. J Hand Surg.

[25] Kuo R, Panchal M, Tanenbaum L, Crues JV (2007). 3.0 Tesla imaging of the musculoskeletal system. JMRI.

[26] Burns JE, Tanaka T, Ueno T, Nakamura T, Yoshioka H (2011). Pitfalls that may mimic injuries of the triangular fibrocartilage and proximal intrinsic wrist ligaments at MR imaging. Radiographics.

[27] Levadoux M, Nguyen MK, Gaillard C (2007). Arthroscopic management of post traumatic wrist sub-acute and chronic disorders. Eur J Orthop Surg Traumatol.

[28] Deniz G, Kose O, Yanik S, Colakoglu T, Tugay A (2014). Effect of untreated triangular fibrocartilage complex (TFCC) tears on the clinical outcome of conservatively treated distal radius fractures. Eur J Orthop Surg Traumatol.

